# Socioeconomic status moderates the association between perceived environment and active commuting to school

**DOI:** 10.11606/S1518-8787.2018052000189

**Published:** 2018-11-14

**Authors:** Alexandre Augusto de Paula da Silva, Rogério César Fermino, Carla Adriane Souza, Alex Vieira Lima, Ciro Romelio Rodriguez-Añez, Rodrigo Siqueira Reis

**Affiliations:** 1 Universidade Federal do Paraná. Programa de Pós-Graduação em Educação Física. Curitiba, PR, Brasil; 2 Pontifícia Universidade Católica do Paraná. Grupo de Pesquisa em Atividade Física e Qualidade de Vida. Curitiba, PR, Brasil; 3 Universidade Tecnológica Federal do Paraná. Programa de Pós-Graduação em Educação Física. Grupo de Pesquisa em Ambiente, Atividade Física e Saúde. Curitiba, PR, Brasil; 4 Pontifícia Universidade Católica do Paraná. Programa de Pós-Graduação em Gestão Urbana. Curitiba, PR, Brasil; 5 Washington University in St. Louis. Brown School. Prevention Research Center. Saint Louis, MO, United States of America

**Keywords:** Adolescent, Parent-Child Relations, Socioeconomic Factors, Social Environment, Transportation

## Abstract

**OBJECTIVE:**

To analyze the moderator effect of socioeconomic status in the association between the perceived environment and active commuting to school.

**METHODS:**

A total of 495 adolescents and their parents were interviewed. Perceived environment was operationalized in traffic and crime safety and assessed with the Neighborhood Environment Walkability Scale. Active commuting was self-reported by the adolescents, categorized in walking, bicycling or skating at least one time/week. Socioeconomic status was used as moderator effect, reported from adolescents' parents or guardians using Brazilian standardized socioeconomic status classification. Analyses were performed with Poisson regression on Stata 12.0.

**RESULTS:**

Prevalence of active commuting was 63%. Adolescents with low socioeconomic status who reported “it is easy to observe pedestrians and cyclists” were more likely to actively commute to school (PR = 1.18, 95%CI 1.03–1.13). Adolescents with low socioeconomic status whose parents or legal guardians reported positively to “being safe crossing the streets” had increased probability of active commuting to school (PR = 1.10, 95%CI 1.01–1.20), as well as those with high socioeconomic status with “perception of crime” were positively associated to the outcome (PR = 1.33, 95%CI 1.03–1.72).

**CONCLUSIONS:**

Socioeconomic status showed moderating effects in the association between the perceived environment and active commuting to school.

## INTRODUCTION

Global estimates suggest eight in every 10 adolescents do not comply with recommendations of daily 60 minutes of moderate-to-vigorous physical activities^1^, similar to those in Brazil^2^. One possibility to increase physical activity levels is the incentive for daily active commuting, characterized by walking or bicycling to school, work or other destinations^3^. In fact, a positive association between active commuting and health indicators, such as body composition and cardiovascular fitness in children and adolescents, has been reported in the literature^3^. However, the percentage of active commuting to school in adolescents varies from 35% to 70%^4^ ^–^ ^7^ in high income countries and between 12% and 70%^8^ ^,^ ^9^ in Brazil. This indicates a high individual and regional variability, such as cities' built environments, policies and social characteristics.

Individual (gender, income)^5^ ^,^ ^6^, psychosocial (social support)^7^ ^,^ ^10^, perceived (aesthetics)^5^ ^,^ ^6^ ^,^ ^11^, and built environment variables (distance to school, number of crossings and residential density)^4^ ^,^ ^12^ are associated to adolescents' active commuting to school. Moreover, evidence suggests that the way parents perceive the environment may also affect the choice of their children's mode of commuting^4^ ^,^ ^12^ ^–^ ^16^. Most perceived environment variables present inconclusive associations with active commuting, especially those related to traffic^17^. Nonetheless, studies investigating such associations were from high income countries, and evidence is lacking in Brazilian populations^17^.

Theoretical models suggest that parents or legal guardians' perception on lack of safety (theft, robbery, and dangerous crossings) may influence their reasoning when allowing the adolescent to actively commute to school^16^ ^,^ ^18^. Even though no studies have been identified where parental socioeconomic status (SES) may be associated to active commuting to school in adolescents, studies with adults show that those with higher income and negative perception of safety are less likely to actively commute in the neighborhood they reside^19^. However, lack of evidence on perceived environment in adolescents and their parents with active commuting to school highlights the need for such investigation. To this moment, no evidence has been provided on the moderator effect that SES may have in the association between the perceived environment and active commuting to school. A better understanding of this relationship can improve the implementation and orientation of strategies to promote active commuting to school by making objective changes to the environment or altering perception of residents.

The aim of this study was to test the moderator effect of the SES in the association between the perception of adolescents and their parents or legal guardians on neighborhood environment and active commuting to school in adolescents.

## METHODS

### Characteristics of the Study and Ethical Aspects

The data used in this study is part of an international, multicenter project conducted in 19 countries (IPEN – International Physical Activity and the Environment Network). In Brazil, data was collected in the city of Curitiba, state of Paraná, between the months of August 2013 and May 2014. This is a cross-sectional study with household and face-to-face interviews. More information about the study is available in the literature^20^. The Project was approved by the Ethics Committee at the Pontifícia Universidade Católica do Paraná (Process 135-945/2012), and adolescents and their parents signed an informed consent.

### Selection of Census Tracts

The 2,395 census tracts in the city were considered primary sampling units and selected according to walkability and income^20^ ^,^ ^21^. Walkability was defined as a combination of characteristics on land-use mix, residential density and street connectivity, being a measure consistently related with physical activity^21^. Land-use mix was determined according to the distribution of five categories (residential, commercial, recreational, educational/cultural, and other). Residential density was calculated by the ratio between residential units and land area. Street connectivity was computed as the density of intersections within a certain census tract. Raw values were normalized, and z-scores calculated.

Aiming to maximize the variability in walkability and income, census tracts were classified in deciles for both variables. Four groups were created to represent locations with: “high walkability and high income”, “high walkability and low income”, “low walkability and high income” and “low walkability and low income”. Eight census tracts were intentionally selected from each group (n = 32 census tracts in total).

### Selection of Households and Participants

Street segments were listed for all blocks within census tracts, the first being located on the southeast extreme of the census tract. Homes were visited in person from the left upper side of the block, clockwise. In case a family refused to participate or there were no adolescents in the household, the next home on the left side was visited.

For each home, one adolescent and one of their parents or legal guardians were selected. The order of selection was: younger females, followed by older males, to allow for an equitable gender selection. In case the selected adolescent refused to participate, another from the same household could be intentionally recruited. According to recommendations of the project protocol, minimum sample should be of 300 adolescents.

Adolescents included were 12–17 years old, residing in the census tract for at least one year from data collection and must be enrolled in school. Those with physical limitations preventing physical activity or cognitive limitations preventing comprehension of the questionnaire were excluded from data collection.

### Data Collection

Twenty-three undergraduate and graduate students were responsible for interviewing families after 12 hours of training. Sessions included selection criteria, how to approach homes and participants, surveys, concepts, reading questions and emphasis on highlighted topics, identification of appropriated answers, and forms and identification of refusal. Simulation of the data collection process was done to ensure understanding about procedures.

### Dependent Variable

Active commuting to school was assessed by the question: “In a regular week, how many days and for how long do you use the following transport mode to go to or come back from school?”. Six options were available: walking, bicycle, skateboard, public transport, school bus or car.

For analysis purpose, the variable “active commuting to school” was operationalized by weekly frequency, in “zero time/week” *versus* “≥ 1 times/week”, independent from time spent in commute. This measure and its operationalization have been used in similar studies^4^ ^,^ ^7^.

### Independent Variables

Neighborhood environment perception of adolescents and their parents or legal guardians was assessed by the Neighborhood Environment Walkability Scale (NEWS). There are two versions of this instrument, one specific for adolescents (NEWS-Youth) and one for adults (NEWS-Adults). Both were translated, adapted and validated to the Brazilian context^22^ ^,^ ^23^.

Eight questions were selected from NEWS and NEWS-Youth, related to perception of safety in traffic, and seven on perception of crime, potentially associated to active commuting to school in adolescents^4^ ^,^ ^16^. The eight questions on traffic safety were: 1) “Is there a lot of traffic in your neighborhood preventing you from walking?”; 2) “Is the traffic speed usually low?”; 3) “Do cars drive by above the speed limit?”; 4) “Is there a lot of smoke/pollution from exhaust fumes?”; 5) “Are the streets well lit at night?”; 6) “Are pedestrians and bicyclists easily seen from inside your home?”; 7) “Are there crosswalks and signals for pedestrians to cross?”; 8) “Do you feel safe crossing the streets in your neighborhood?”. The seven questions on crime safety were: 1) “Is there a lot of crime in your neighborhood?”; 2) “Does crime make it unsafe to walk during the day?”; 3) “Does crime make it unsafe to walk at night?”; 4) “Do you worry about being alone around your neighborhood?”; 5) “Do you worry about being with a friend around your home?”; 6) “Do you worry about being around your home because you are afraid of being robbed?”; 7) “Do you worry about being in parks around your home because you are afraid of being robbed?”. Parents were questioned about environment perception related to safety of their children (Tables 1 and 2). Answers were in a four-point Likert scale: “totally disagree”, “disagree a little”, “agree a little” and “totally agree”. For analysis purpose, the options “totally disagree” and “disagree a little” were grouped and operationalized as “no” (code: 0). Options “agree a little” and “totally agree” were grouped and operationalized as “yes” (code: 1), which represented, respectively, lack and presence of attribute.

**Table 1:** Descriptive characteristics of participants per socioeconomic status. Curitiba, state of Paraná, Brazil, 2013-2014. (n = 495)

Variable	Category	Low (n = 195; 39.4%)		High (n = 300; 60.6%)		p	Total (n = 495; 100%)	
		n	%	n	%		n	%
Adolescents								
Gender	Male	99	50.8	145	48.3	0.596^h^	244	49.3
	Female	96	49.2	155	51.7		251	50.7
Age group (years)	12-13	86	44.1	119	39.7	0.050^g^	205	41.4
	14-15	73	37.4	96	32.0		169	34.1
	16-17	36	18.5	85	28.3		121	24.4
Nutritional status	Normal weight	106	57.6	167	60.5	0.535^h^	273	59.3
	Overweight	78	42.4	109	39.5		187	40.7
Perception of time to walk to school (minutes)	≤ 10	83	43.9	63	21.1	< 0.001^g^	146	29.9
	11-20	43	22.8	80	26.8		123	25.2
	21-30	26	13.8	43	14.4		69	14.1
	≥ 31	37	19.6	113	37.8		150	30.7
Leisure time, moderate to vigorous physical activity (min/week)	< 300	110	56.4	188	62.7	0.165^h^	298	60.2
	≥ 300	85	43.6	112	37.3		197	39.8
Active commuting to school (≥ 1 times/week)^a^	No	37	19.7	143	47.8	< 0.001^h^	180	37.0
	Yes	151	80.3	156	52.2		307	63.0
Period spend in school/studying	Morning	105	55.6	217	73.1	< 0.001^h^	322	66.3
	Afternoon/Evening	84	44.4	84	26.9		164	33.7
Parents or legal guardian	Father	15	7.7	55	18.3	< 0.001^g^	70	14.1
	Mother	151	77.4	220	73.3		371	74.9
	Other^b^	29	14.9	25	8.3		54	10.9
Car ownership	No	99	50.8	5	1.7	< 0.001^h^	104	21.0
	Yes	96	49.2	295	98.3		391	79.0
Active commuting through the neighborhood (≥ 1 times/ week, ≥ 10 min)^c^	No	52	26.7	145	48.3	< 0.001^h^	197	39.8
	Yes	143	73.3	155	51.7		298	60.2
Total leisure time physical activity (min/week)	< 150	166	85.6	208	69.6	< 0.001^h^	374	75.9
	≥ 150	28	14.4	91	30.4		119	24.1
Adolescents' perception								
Traffic								
Is there a lot of traffic in your neighborhood, for you to walk?	No	102	52.3	173	57.7	0.241^h^	275	55.6
	Yes	93	47.7	127	42.3		220	44.4
Is the speed of traffic usually low?	No	96	49.7	143	48.1	0.730^h^	239	48.8
	Yes	97	50.3	154	51.9		251	51.2
Do drivers drive over the speed limit?	No	50	25.8	91	30.4	0.263^h^	141	28.6
	Yes	144	74.2	208	69.6		352	71.4
Is there a lot of smoke/pollution from exhaust fumes?	No	100	51.3	166	55.3	0.377^h^	266	53.7
	Yes	95	48.7	134	44.7		229	46.3
Are streets well lit at night?	No	67	34.4	125	41.7	0.103^h^	192	38.8
	Yes	128	65.6	175	58.3		303	61.2
Can pedestrians and bicyclists be seen by people from inside their homes?	No	54	27.7	83	27.7	0.995^h^	137	27.7
	Yes	141	72.3	217	72.3		358	72.3
Are there crosswalks and signals to help pedestrians cross the streets?	No	84	43.1	129	43.0	0.987^h^	213	43.0
	Yes	111	56.9	171	57.0		282	57.0
Do you feel safe when crossing the streets of your neighborhood?	No	89	45.6	117	39.0	0.143^h^	206	41.6
	Yes	106	54.4	183	61.0		289	58.4
Crime								
Is there a lot of crime in your neighborhood?	No	86	44.1	137	45.7	0.733^h^	223	45.1
	Yes	109	55.9	163	54.3		272	54.9
Does crime make it unsafe to walk during the day?	No	120	61.5	198	66.0	0.312^h^	318	64.2
	Yes	75	38.5	102	34.0		177	35.8
Does crime make it unsafe to walk at night?	No	23	11.8	34	11.3	0.875^h^	57	11.5
	Yes	172	88.2	266	88.7		438	88.5
Do you worry about being alone around your home?	No	140	71.8	216	72.2	0.914^h^	356	72.1
	Yes	55	28.2	83	27.8		138	27.9
Do you worry about being with a friend around your home?	No	152	77.9	233	77.7	0.941^h^	385	77.8
	Yes	43	22.1	67	22.3		110	22.2
Do you worry about being around your home because you are afraid of being robbed?	No	93	47.7	132	44.0	0.420^h^	225	45.5
	Yes	102	52.3	168	56.0		270	54.5
Do you worry about being in parks around your home because you are afraid of being robbed?	No	85	43.6	136	45.3	0.703^h^	221	44.6
	Yes	110	56.4	164	164		274	55.4
Sum of items for traffic and crime								
Score for perception of safety related to traffic	Tertile 1	100	51.8	148	50.0	0.194^g^	248	50.7
	Tertile 2	47	24.4	52	17.6		99	20.2
	Tertile 3^d^	46	23.8	96	32.4		142	29.0
Score for perception of safety related to crime	Tertile 1	85	43.6	138	46.2	0.976^g^	223	45.1
	Tertile 2	82	42.1	111	37.1		193	39.1
	Tertile 3	28	14.4	50	16.7		78	15.8
Score for overall perception of safety (traffic + crime)	Tertile 1	85	44.0	124	42.0	0.478^g^	209	42.8
	Tertile 2	49	25.4	70	23.7		119	24.4
	Tertile 3	59	30.6	101	34.2		160	32.8
Parents or legal guardian's perception								
Traffic								
Is there a lot of traffic in the neighborhood for the adolescent to walk?	No	100	51.3	139	46.3	0.282^h^	239	48.3
	Yes	95	48.7	161	53.7		256	51.7
Is the traffic speed usually low?	No	116	59.5	185	61.7	0.639^h^	301	60.8
	Yes	79	40.5	115	38.3		194	39.2
Do drivers drive over the speed limit?	No	39	20.0	86	28.8	0.029^h^	125	25.3
	Yes	156	80.0	213	71.2		369	74.7
Is there a lot of smoke/pollution from exhaust fumes?	No	107	54.9	180	60.0	0.259^h^	287	58.0
	Yes	88	45.1	120	40.0		208	42.0
Are streets well lit at night?	No	79	40.5	150	50.0	0.039^h^	229	46.3
	Yes	116	59.5	150	50.0		266	53.7
Can pedestrians and bicyclists be seen by people from inside their homes?	No	65	33.3	129	43.0	0.031^h^	194	39.2
	Yes	130	66.7	171	57.0		301	60.8
Are there crosswalks and signals to help pedestrians cross the streets?	No	109	55.9	154	51.3	0.320^h^	263	53.1
	Yes	86	44.1	146	48.7		232	46.9
Do you think it is safe for the adolescent to cross the streets of your neighborhood?	No	130	66.7	181	60.3	0.154^h^	311	62.8
	Yes	65	33.3	119	39.7		184	37.2
Crime								
Is there a lot of crime in your neighborhood?	No	73	37.4	113	37.7	0.959^h^	186	37.6
	Yes	122	62.6	187	62.3		309	62.4
Does crime make it unsafe to walk during the day?	No	86	44.1	113	37.7	0.154^h^	199	40.2
	Yes	109	55.9	187	62.3		296	59.8
Does crime make it unsafe to walk at night?	No	14	7.2	19	6.3	0.712^h^	33	6.7
	Yes	181	92.8	281	93.7		462	93.3
Do you worry about the adolescent being alone around your home?	No	88	45.4	146	48.7	0.472^h^	234	47.4
	Yes	106	54.6	154	51.7		260	52.6
Do you worry about the adolescent being with a friend around your home?	No	89	45.6	146	48.7	0.510^h^	235	47.5
	Yes	106	54.4	154	51.3		260	52.5
Do you worry about the adolescent being around your home because you are afraid he/she will be robbed?	No	30	15.4	48	16.0	0.854^h^	78	15.8
	Yes	165	84.6	252	84.0		417	84.2
Do you worry about the adolescent being in parks around your home because you are afraid he/she will be robbed?	No	18	9.2	45	15.0	0.060^h^	63	12.7
	Yes	177	90.8	255	85.0		432	87.3
Sum of items for traffic and crime								
Score for perception of safety related to traffic	Tertile 1	99	50.8	134	44.8	0.250^g^	233	47.2
	Tertile 2	64	32.8	110	36.8		174	35.2
	Tertile 3^d^	32	16.4	55	18.4		87	17.6
Score for perception of safety related to crime	Tertile 1	82	42.3	114	38.0	0.638^g^	196	39.7
	Tertile 2	70	36.1	124	41.3		194	39.3
	Tertile 3^e^	42	21.6	62	20.7		104	21.1
Score for overall perception of safety (traffic + crime)	Tertile 1	70	36.1	105	35.1	0.785^g^	175	35.5
	Tertile 2	69	35.6	118	39.5		187	37.9
	Tertile 3f	55	28.4	76	25.4		131	26.6

a Walking, using bicycle or skateboard to go or return from school . 1 time/week.

b Grandmother: 4.0%; grandfather: 1.6%; aunt: 1.0%; uncle: 0.8%; other: 3.4% (brother, stepfather, etc.).

c Walking, using bicycle to commute through the streets of the neighborhood, at least one day a week, for at least 10 consecutive minutes (. 1 times/week, . 10 min).

d Better perception of safety related to traffic.

e Better perception of safety related to crime.

f Better overall perception of safety (traffic + crime).

g Value for chi-squared test for linear tendency.

h Value for chi-squared test for heterogeneity.

**Table 2:** Bivariate and multivariate association between environment perception by adolescents and active commuting to school per socioeconomic status. Curitiba, state of Paraná, Brazil, 2013-2014. (n = 495)

Variable		Low						High					
		n	%	Crude		Adjusted^d^		n	%	Crude		Adjusted^d^	
				PR	95%CI	PR	95%CI			PR	95%CI	PR	95%CI
Traffic perception													
Is there a lot of traffic in the neighborhood for the adolescent to walk?													
	No	84	83.1	1		1		87	50.5	1		1	
	Yes	67	77.0	0.92	0.79-1.08	0.96	0.86-1.07	69	54.3	1.07	0.79-1.45	0.96	0.74-1.26
Is the speed of traffic usually low?													
	No	70	76.0	1		1		77	53.8	1		1	
	Yes	79	84.0	1.10	0.94-1.29	1.05	0.94-1.16	78	50.9	0.94	0.74-1.20	1.04	0.83-1.30
Do drivers drive over the speed limit?													
	No	36	76.6	1		1		45	50.0	1		1
	Yes	114	81.4	1.06	0.89-1.26	1.10	0.97-1.24	110	52.8	1.05	0.80-1.39	0.89	0.69-1.15
Is there a lot of smoke/pollution from exhaust fumes?													
	No	75	78.1	1		1		78	47.2	1		1	
	Yes	76	82.6	1.05	0.92-1.20	1.03	0.94-1.13	78	58.2	1.23	0.96-1.56	1.10	0.91-1.33
Are streets well lit at night?													
	No	54	81.2	1		1		62	49.6	1		1
	Yes	97	79.5	0.97	0.85-1.10	0.99	0.89-1.10	94	54.0	1.08	0.91-1.29	0.94	0.78-1.15
Can pedestrians and bicyclists be seen by people from inside their homes?													
	No	36	66.6	1		1		47	57.3	1		1	
	Yes	115	85.8	**1.29**	**1.05-1.57**	**1.18^e^**	**1.03-1.36**	109	50.2	0.87	0.70-1.09	0.86	0.71-1.04
Are there crosswalks and signals to help pedestrians cross the streets?													
	No	62	76.5	1		1		58	44.9	1		1
	Yes	89	83.1	1.08	0.94-1.25	1.01	0.91-1.13	98	57.6	1.28	0.98-1.66	1.08	0.88-1.33
Do you think it is safe for you to cross the streets of your neighborhood?													
	No	71	82.5	1		1		60	51.2	1		1	
	Yes	80	78.4	0.95	0.80-1.12	0.95	0.85-1.07	96	52.7	1.02	0.80-1.30	0.99	0.81-1.22
Crime perception													
Is there a lot of crime in your neighborhood?													
	No	66	78.5	1		1		73	53.6	1		1	
	Yes	85	81.7	1.04	0.88-1.21	1.08	0.96-1.20	83	50.9	0.94	0.76-1.17	0.95	0.80-1.13
Does crime make it unsafe to walk during the day?													
	No	93	81.5	1		1		104	52.7	1		1
	Yes	58	78.3	0.96	0.83-1.11	1.03	0.94-1.12	52	50.9	0.96	0.77-1.20	0.97	0.75-1.26
Does crime make it unsafe to walk at night?													
	No	19	82.6	1		1		21	61.7	1		1	
	Yes	132	80.0	0.96	0.77-1.20	1.05	0.93-1.19	135	50.9	0.82	0.58-1.16	0.85	0.65-1.12
Do you worry about being alone around your home?													
	No	114	84.4	1		1		109	50.4	1		1
	Yes	37	69.8	0.83	0.69-0.97	0.93	0.80-1.08	46	56.1	1.11	0.86-1.42	0.97	0.79-1.20
Do you worry about being with a friend around your home?													
	No	117	80.6	1		1		122	52.3	1		1	
	Yes	34	79.0	0.97	0.82-1.16	0.96	0.84-1.10	34	51.5	0.98	0.75-1.28	0.90	0.71-1.14
Do you worry about being around your home because you are afraid of being robbed?													
	No	73	82.9	1		1		67	50.7	1		1
	Yes	78	78.0	0.94	0.84-1.04	1.02	0.95-1.10	89	53.2	1.04	0.85-1.28	1.10	0.93-1.31
Do you worry about being in parks around your home because you are afraid of being robbed?													
	No	61	76.2	1		1		73	54.0	1		1	
	Yes	90	83.3	1.09	0.96-1.24	1.10	0.99-1.24	83	50.6	0.93	0.73-1.18	0.92	0.74-1.14
Sum of items for traffic and crime													
Score for perception of safety related to traffic													
	Tertile 1	73	76.0	1		1		76	51.3	1		1	
	Tertile 2	41	87.2	1.14	0.97-1.34	1.12	0.96-1.31	35	67.3	1.31	0.98-1.73	1.17	0.92-1.47
	Tertile 3^a^	35	81.4	1.07	0.91-1.24	0.94	0.83-1.07	43	45.2	0.88	0.65-1.18	0.89	0.68-1.15
Score for perception of safety related to crime													
	Tertile 1	65	78.3	1		1		73	53.2	1		1	
	Tertile 2	65	82.2	1.05	0.88-1.24	0.97	0.88-1.08	53	47.7	0.89	0.70-1.14	0.95	0.78-1.16
	Tertile 3^b^	21	80.7	1.03	0.84-1.26	0.93	0.82-1.05	29	58.0	1.08	0.84-1.40	1.04	0.79-1.37
Score for overall perception of safety (traffic + crime)													
	Tertile 1	65	79.2	1		1		66	53.2	1		1	
	Tertile 2	39	79.5	1.00	0.81-1.23	1.02	0.92-1.12	34	48.5	0.91	0.67-1.23	1.02	0.77-1.34
	Tertile 3^c^	45	81.8	1.03	0.89-1.19	0.96	0.87-1.06	53	53.0	0.99	0.79-1.23	1.07	0.86-1.32

a Better perception of safety related to traffic.

b Better perception of safety related to crime.

c Better overall perception of safety (traffic + crime).

d Adjusted for variables which presented p < 0.20 in the bivariate analysis with active commuting (adolescents: gender, age group, perception of time spent to walk to school, leisure time moderate to vigorous physical activity, period spent in school; guardians: car ownership).

Bolded values for p < 0.05

Based on the sum of individual items, a safety perception score was computed for traffic and crime. Some variables were recoded (0 to 1) to better represent the perception of the environment (safer). On traffic perception, questions 1, 3 and 4 were recoded while all questions on crime perception were recoded. Three safety perception indicators were operationalized based on the scores: 1) traffic; 2) crime and 3) general safety (traffic + crime), categorized in tertiles, indicating “low”, “medium” and “high” environment perception.

### Covariables

The following variables were included as covariables: gender, age group, perception of time spent walking to school, moderate-to-vigorous physical activity in leisure time and period of the day spent in school. Parents or legal guardians' variables included were: car ownership, active commuting in the neighborhood and leisure time physical activity.

The adolescents' gender was observed (“male”, “female”) and their age classified into three age groups (“12–13 years”, “14–15 years” and “16–17 years”). Body mass (kg) and height (cm) were measured and used to calculate body mass index (BMI) and estimated nutritional status, categorized in “normal weight” (low weight and normal weight) and “overweight” (overweight and obese), specific for Brazilian adolescents. Perceived distance to school was assessed with the question: “How long does/would it take for you to walk to school? (even if you don't walk)”^4^ ^,^ ^12^. The answers were grouped into four categories: “≤ 10 min”, “11–20 min”, “21–30 min” and “≥ 31 min”. Leisure time moderate-to-vigorous physical activity, in a regular week, was self-reported as the weekly frequency and duration of any type of activity in that intensity (swimming, sports, dance, races, gymnastics, walking, skateboarding, etc.)^24^. The volume of physical activity was classified in: “< 300 min/week” and “≥ 300 min/week”. The period spent in school was categorized as “morning” or “afternoon/evening”.

An adult considered parent or a legal guardian for the adolescent participated in the survey. For the analysis, the options “grandmother”, “grandfather”, “uncle”, “aunt” and “other” were operationalized as “other”. Car ownership was assessed by the question: “How many motor vehicles (cars, motorcycles, etc.) do you own?”. The answer was classified as “no” (zero vehicles) and “yes” (≥ 1 vehicles). Active commuting was assessed by the question: “Do you walk or bike for at least 10 consecutive minutes to go from one place to another in the neighborhood?” (“no”, “yes”). Total leisure time physical activity was assessed by the long version of the International Physical Activity Questionnaire^25^, and its score calculated by the equation: [walking + moderate activity + (vigorous × 2)], and then classified in two categories: “< 150 min/week” and “≥ 150 min/week”.

### Moderator Variable

The SES was assessed by a standard questionnaire which considers a number of domestic appliances, presence of a housekeeper, and education of the financial provider for the household^26^, later classified in seven levels. For the analysis purpose of the moderator effect, participants were classified in two SES: “low” (classes C+D+E) and “high” (classes A+B).

### Data Analysis

Poisson regression was used to test the association between the perceived environment for adolescents and their parent or legal guardians and active commuting to school. In order to test the possible moderator effect, the SES was included in the model (“low” *versus* “high”). After bivariate analysis, the multivariate model was adjusted for individual variable which presented p < 0.20 in the bivariate model (adolescents: gender, age group, perception of time spent to walk to school, leisure time moderate-to-vigorous physical activity, period spent in school; parents: car ownership). Data were analyzed in Stata 12.0 and significance level was kept at 5%.

## RESULTS

A total of 495 adolescents were interviewed (50.7% girls), as well as their respective parents or legal guardians (74.9% mothers). Refusal rate of participation was 16% (n = 94), similar between neighborhood income. Frequency of active commuting was 63.0%, being higher among adolescents of low SES (80.3% *versus* 52.2%, p < 0.001).

Most adolescents were within the 12-13 years-old age group (41.4%), had normal nutritional status (59.3%), their perceived distance walking to school was ≥ 31 minutes (30.7%), leisure time moderate-to-vigorous physical activity was < 300 min/week (60.2%), and studied during the morning period (66.3%) (Table 1). For parents or legal guardians, a higher proportion owned at least one motor vehicle (79.0%), were active commuters through the neighborhood (60.2%), and practiced < 150 min/week of leisure time moderate-to-vigorous physical activities (75.9%). Among individuals of low SES, there was higher proportion of other relatives (grandmother, grandfather, aunt, uncle, brother) as responsible for the adolescent (p < 0.001). The high SES was associated to car ownership and with ≥ 150 min/week of total leisure time physical activity (p < 0.001) (Table 1). For parents or legal guardians, the perception of neighborhood characteristics such as “drivers drive over the speed limit”, “streets are well lit at night” and “pedestrians and bicyclists can be easily seen from inside their homes”, was positively associated to low SES (p < 0.05) (Table 1).

In the bivariate analysis for low SES adolescents, the fact that it was possible to “see pedestrians and bicyclists from inside their homes” was positively associated to active commuting to school (PR = 1.29, 95%CI 1.05–1.57), while “concern of being alone around the home” was inversely associated to the outcome (PR = 0.83, 95%CI 0.69–0.97) (Table 2). On parents or legal guardians' perception, those of high SES perceived “a lot of smoke and pollution” (PR = 1.20, 95%CI 1.02–1.41) and that “streets were well lit at night” (PR = 1.30, 95%CI 1.09–1.55) were positively associated to active commuting to school (Table 3).

**Table 3:** Bivariate and multivariated association between the environment perception by parents or legal guardian and active commuting to school, by socioeconomic level. Curitiba, state of Paraná, Brazil, 2013-2014. (n = 495)

Variable		Low						High					
		n	%	Crude		Adjusted		n	%	Crude		Adjusted	
				PR	95%CI	PR	95%CI			PR	95%CI	PR	95%CI
Traffic perception													
Is there a lot of traffic in the neighborhood for the adolescent to walk?													
	No	78	80.4	1		1		71	51.0	1		1
	Yes	73	80.2	0.99	0.84-1.17	1.02	0.90-1.15	85	53.1	1.04	0.83-1.30	1.03	0.87-1.22
Is the speed of traffic usually low?													
	No	89	80.9	1		1		94	50.8	1		1	
	Yes	62	79.4	0.98	0.85-1.12	0.92	0.82-1.04	62	54.3	1.07	0.83-1.36	1.00	0.81-1.23
Do drivers drive over the speed limit?													
	No	28	73.6	1		1		51	59.3	1		1
	Yes	123	82.0	1.11	0.89-1.38	1.10	0.96-1.27	104	49.0	0.82	0.67-1.00	0.86	0.69-1.07
Is there a lot of smoke/pollution from exhaust fumes?													
	No	81	79.4	1		1		87	48.3	1		1	
	Yes	70	81.4	1.02	0.85-1.22	0.98	0.87-1.11	69	57.9	**1.20**	**1.02-1.41**	1.13	0.97-1.31
Are streets well lit at night?													
	No	56	72.7	1		1		68	45.3	1		1
	Yes	95	85.5	1.17	0.98-1.40	1.14	0.98-1.32	88	59.0	**1.30**	**1.09-1.55**	1.12	0.96-1.30
Can pedestrians and bicyclists be seen by people from inside their homes?													
	No	50	78.1	1		1		63	49.2	1		1	
	Yes	101	81.4	1.04	0.89-1.22	1.01	0.90-1.13	93	54.3	1.10	0.88-1.38	1.03	0.85-1.25
Are there crosswalks and signals to help pedestrians cross the streets?													
	No	84	78.5	1		1		77	50.0	1		1
	Yes	67	82.7	1.05	0.93-1.18	0.99	0.88-1.11	79	54.4	1.08	0.89-1.32	0.99	0.86-1.14
Do you think it is safe for the adolescent to cross the streets of your neighborhood?													
	No	97	77.6	1		1		90	50.0	1		1	
	Yes	54	85.7	1.10	0.96-1.26	**1.10**	**1.01-1.20**	66	55.4	1.10	0.91-1.34	1.04	0.83-1.29
Crime perception													
Is there a lot of crime in your neighborhood?													
	No	63	87.5	1		1		50	44.6	1		1	
	Yes	88	75.8	0.86	0.75-1.00	0.94	0.83-1.06	106	56.6	1.26	0.97-1.65	**1.33**	**1.03-1.72**
Does crime make it unsafe to walk during the day?													
	No	66	81.4	1		1		60	53.1	1		1
	Yes	85	79.4	0.97	0.82-1.15	0.93	0.85-1.03	96	51.6	0.97	0.77-1.22	0.96	0.76-1.21
Does crime make it unsafe to walk at night?													
	No	13	92.8	1		1		10	52.6	1		1	
	Yes	138	79.3	0.85	0.71-1.02	0.98	0.86-1.13	146	52.1	0.99	0.55-1.75	1.07	0.66-1.73
Do you worry about the adolescent being alone around your home?													
	No	66	78,5	1		1		78	53,7	1		1
	Yes	84	81,5	1,03	0,93-1,15	1.03	0.95-1.11	78	50,6	0,94	0,78-1,12	1.01	0.85-1.21
Do you worry about the adolescent being with a friend around your home?													
	No	67	78,8	1		1		72	49,6	1		1	
	Yes	84	81,5	1,03	0,90-1,18	1.01	0.93-1.10	84	54,5	1,09	0,91-1,32	1.07	0.91-1.26
Do you worry about the adolescent being around your home because you are afraid he/ she will be robbed?													
	No	22	81,4	1		1		26	54,1	1		1
	Yes	129	80,1	0,98	0,81-1,19	0.90	0.76-1.07	130	51,7	0,95	0,73-1,24	1.01	0.79-1.28
Do you worry about the adolescent being in parks around your home because you are afraid he/she will be robbed?													
	No	13	81,2	1		1		18	40,0	1		1	
	Yes	138	80,2	0,98	0,78-1,24	0.80	0.64-1.00	138	54,3	1,35	0,94-1,96	1.16	0.85-1.59
Sum of items for traffic and crime													
Score for perception of safety related to traffic													
	Tertile 1	75	77,3	1		1		63	47,3	1		1	
	Tertile 2	48	81,3	1,05	0,91-1,20	0.98	0.90-1.07	62	56,3	1,18	0,92-1,53	1.09	0.88-1.36
	Tertile 3^a^	28	87,5	1,13	0,98-1,30	1.08	0.93-1.26	30	54,5	1,15	0,91-1,44	0.98	0.79-1.22
Score for perception of safety related to crime													
	Tertile 1	64	80,0	1		1		62	54,3	1		1	
	Tertile 2	53	77,9	0,97	0,84-1,12	1.00	0.91-1.08	64	52,0	0,95	0,77-1,18	0.94	0.78-1.13
	Tertile 3^b^	33	84,6	1,05	0,89-1,25	1.08	0.97-1.21	30	48,3	0,88	0,68-1,16	0.83	0.64-1.08
Score for overall perception of safety (traffic + crime)													
	Tertile 1	55	79,7	1		1		55	52,3	1		1	
	Tertile 2	51	78,4	0,98	0,81-1,18	0.94	0.82-1.07	60	51,2	0,97	0,77-1,24	1.06	0.86-1.30
	Tertile 3^c^	44	83,2	1,04	0,86-1,25	1.04	0.90-1.20	40	52,6	1,00	0,82-1,21	0.90	0.75-1.09

a Better perception of safety related to traffic.

b Better perception of safety related to crime.

c Better overall perception of safety (traffic + crime).

d Adjusted for variables which presented p < 0.20 in the bivariate analysis with active commuting (adolescents: gender, age group, perception of time spent to walk to school, leisure time moderate to vigorous physical activity, period spent in school; guardians: car ownership).

Bolded values for p < 0.05

After adjusting for confounding variables, only the perception of being possible to “see pedestrians and bicyclists from inside their homes” remained positively associated to active commuting to school among low SES adolescents (PR = 1.18, 95%CI 1.03–1.36) (Table 2). For parents or legal guardians' perception, among those of low SES, the perception of “being safe for adolescents to cross the street in the neighborhood” was positively associated to adolescents' active commuting to school (PR = 1.10, 95%CI 1.01–1.20). For those of high SES, the “perception of too much crime in the neighborhood” was positively associated to active commuting (PR = 1.33, 95%CI 1.03–1.72) (Table 3).

## DISCUSSION

This is the first study looking to identify the association between the perceived environment of the neighborhood by adolescents and their parents or legal guardians and active commuting to school in Brazilian adolescents. Moreover, the first to explore the moderator effect of the SES in this association. The methodology allowed to explore how different perceptions of the environment may affect the behavior of adolescents in active commuting to school, that being the strength of this study. Previous studies have tested the association between the isolated environment perception of adolescents and their guardians and active commuting to school^17^.

The proportion of active commuting to school was higher among adolescents of low SES (80.3% *versus* 52.2%, p < 0.001). The premise that the environment perception by guardians could, somehow, influence the decision to allow their children to commute actively to school, was not confirmed in most associations tested independent from SES.

In the present study the perception of being possible to “see pedestrian and bicyclists from inside the home” increased in 18% the probability of active commuting to school for low SES adolescents. Only one study testing this association with a similar variable was found^11^. Evenson et al.^11^ found a negative association between the fact of “seeing people in the neighborhood” and active commute to school in girls in the United States, such results being different from the present study. This difference may be partly explained by an increased concern by guardians to girls' exposure to more dangerous environments^27^. However, exploratory analyses did not identify an association between the perception of guardians and adolescents' gender (data not presented). Moreover, it is important to highlight the measurement and how participants were asked about it “being possible to see other people in the community” (“from inside their homes” *versus* “see people in the neighborhood in general”). This could, somehow, modify the perception of those related to this characteristic^11^. In the present study, the moderator effect of low SES in the association found may be explained by the fact that adolescents of lower SES would find active commute a necessity, once it includes an economic cost and fewer access to motorized means of transportation. This characteristic could increase the perception of being possible to see people in the neighborhood^28^.

For parents or legal guardians of low SES, perception of safety related to crossings may increase in 10% the probability of adolescents engaging in active commute to school. Only one study found similar results^15^. Other evidence has pointed to the positive association between better general characteristics of traffic (signals, signs indicating speed control, traffic volume, etc.) and active commute to school^13^ ^,^ ^14^ ^,^ ^16^ ^,^ ^17^ ^,^ ^29^ ^,^ ^30^, these being similar to what was found. In fact, some studies suggest that better traffic conditions could increase the perception of safety of individuals, facilitating active commuting^13^ ^,^ ^30^. The diminished access to motor vehicles by guardians of low SES (49.2% *versus* 98.3%, p < 0.001) may contribute to more active commuting through neighborhood streets (73.3% *versus* 51.7%, p < 0.001). This characteristic, associated to the impossibility of guardians to pay for public or private transport for their children to go to school, would allow better knowledge of neighborhood characteristics^28^.

Parents or legal guardians of high SES had the perception of crime positively associated to active commute to school (PR = 1.33, 95%CI 1.03–1.72). Several studies have explored the association between the different variables for perception of crime and active commute to school and have not found significant association between the variables^4^ ^,^ ^17^ ^,^ ^29^. In spite of similarity to this study in several of the associations tested, except for the presence of crime in the neighborhood, there is inconsistency in the results of studies looking into this variable. For example, a study in Nairobi (Kenya) did not find associations between the perception of crime and active commuting^15^. The positive association found in this study is different than what is expected and reported in the literature, allowing us to hypothesize this to be a spurious association due to characteristics of the sample.

Most variables of environment perception explored in this study were not associated to active commuting. However, it is important to highlight that these results are similar to those verified in the literature^17^. D'Haese et al.^17^ state that results from cross-sectional studies tend to present a positive association between few variables for perception of traffic and active commute to school. Nonetheless, the same authors affirm that such associations are not verified when considering perception of crime^17^. The lack of associations between perception of traffic and crime and active commute to school in adolescents in this study may be due to the fact that this behavior is influenced by other predictors in the neighborhood^17^. For example, studies show that adolescents living in high walkability neighborhoods and access to services are more likely to actively commute to school when compared to adolescents living in antagonist neighborhoods^16^ ^,^ ^17^.

Some limitations must be considered when interpreting and extrapolating results. The sample is not representative of adolescents in the city, once participants were selected from intentionally selected census tracts to generate contrasts for walkability and income. It is likely that associations tested in this study present different results if census tracts with less variability were considered. Moreover, other characteristics were not considered in this study, as parents' (“father” or “mother”) perceptions and environmental (walkability, aesthetics, access to services, mixed land use) ones, which can be associated to the outcome^16^ ^,^ ^17^. At last, the cross-sectional design does not allow for a causal relationship to be determined.

In conclusion, the SES presented a moderating effect in the association between the perceived environment by adolescents and their parents or legal guardians, with active commuting to school. For adolescents with low SES, the fact that they can “see pedestrians and cyclists from inside their homes” was positively associated with active commute to school. For parents or legal guardian with low SES, perception of safety to “cross the streets in the neighborhood” was positively associated with active commute, while for those with high SES, perception of crime was associated to the outcome.

The improvement in the conditions of neighborhood safety may positively influence the perception of safety in adolescents and their parents or legal guardians. Presence of traffic signals in commuting routes to school and in school surroundings could improve the probability of active commuting to school^18^. These results suggest administrators should implement interventions based on the modification of environmental characteristics to favor active commuting. Future studies must analyze the effect of such environmental modifications in individual perception of safety and the relation of this effect with active commute to school.
